# Molecular Insights Shaping the Design of Metal–Organic Framework-Based Electrolytes

**DOI:** 10.1021/acs.chemmater.6c01203

**Published:** 2026-06-19

**Authors:** Shoushou He, Julius J. Oppenheim, Keiichiro Maegawa, Zhentao Yang, Yunyao Xu, Ann E. McDermott, Mircea Dincă

**Affiliations:** † Department of Chemistry, 6740 Princeton University, Princeton, New Jersey 08540, United States; ‡ Department of Chemistry, 5798 Columbia University, New York, New York 10027, United States

## Abstract

Understanding the molecular determinants of Li^+^ transport in quasi-solid-state electrolytes (QSSEs) is critical for the design of next-generation energy storage materials. Here, we investigate Li^+^ conduction in an isoreticular series of anionic metal–organic frameworks (MOFs), Li_3_[(Cu_4_Cl)_3_L_8_] (L^3–^ = linker), to disentangle the effects of pore size and linker functionality on ion conductivity. Contrary to expectation based on previous studies, despite their structural differences, the MOFs show pore size-independent Li^+^ conductivity, whereas the linker functional groups can impose an effect. Using ^7^Li magic-angle-spinning solid-state nuclear magnetic resonance, we identify the major Li^+^ conducting species residing in the open pore channels, which, upon the addition of propylene carbonate, display liquid-like transport with low activation energies. The similar local environments and liquid-like dynamics of these species give rise to conductivities on the order of 10^–5^ S/cm across the isoreticular series, though the interactions between tetrazolate nitrogens in linkers and Li^+^ restrict ion mobility, leading to a modest decrease in conductivity. This study provides molecular-level insights into Li^+^ transport in anionic MOF-based electrolytes and thus establishes the design principles for developing efficient QSSEs.

## Graphical abstract

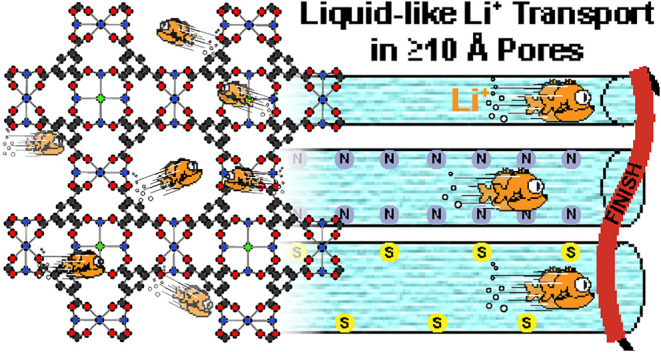

Quasi-solid-state electrolytes (QSSEs) are an advantageous class of electrolytes as they combine the molecular tunability of liquid electrolytes with the processability of solid-state electrolytes.^1–4^ Of these, anionic metal–organic frameworks (MOFs) stand out due to their combination of intrinsic porosity, crystallinity, synthetic flexibility, and high transference numbers.^5–8^ These features enable a wide variety of cations to conduct with high efficiency.^9,10^ Despite these unique advantages, we still lack a fundamental understanding of how the framework structure (including pore size, functional group, ion concentration, and ion valency) affects ion conductivity, hindering the design of MOFs as QSSEs.^11–15^ To bridge this knowledge gap, we study the molecular factors governing Li^+^ transport in a series of isoreticular anionic MOFs, specifically targeting the effects of pore size and functional group identity.

The anionic Li_3_[(Cu_4_Cl)_3_L_8_] (L^3–^ = linker) MOF series offers a uniquely controlled platform for probing Li^+^ transport on a molecular level,^16–18^ as varying the linkers tunes pore size and functionality while preserving the same (3,8)-connected sodalite-type topology (Figure 1) — a rare feature since linker variation in MOFs typically alters connectivity, hence framework topology. These frameworks feature an 8-connected [Cu_4_Cl]^7+^ secondary building unit (SBU) composed of a square plane of Cu^2+^ ions surrounding an interstitial chloride that imparts the framework’s anionic charge (Figure 1), balanced by extra-framework cations. The [Cu_4_Cl]^7+^ SBUs occupy the vertices of truncated octahedra and are bridged by planar tritopic linkers on the octahedral faces, yielding octahedral cages that connect to form open channels along all three crystallographic directions defined by the pore-limiting window diameter (d_Cu···Cu_) (Figure 1). These channels serve as the Li^+^ transport pathways.

**Figure 1:**
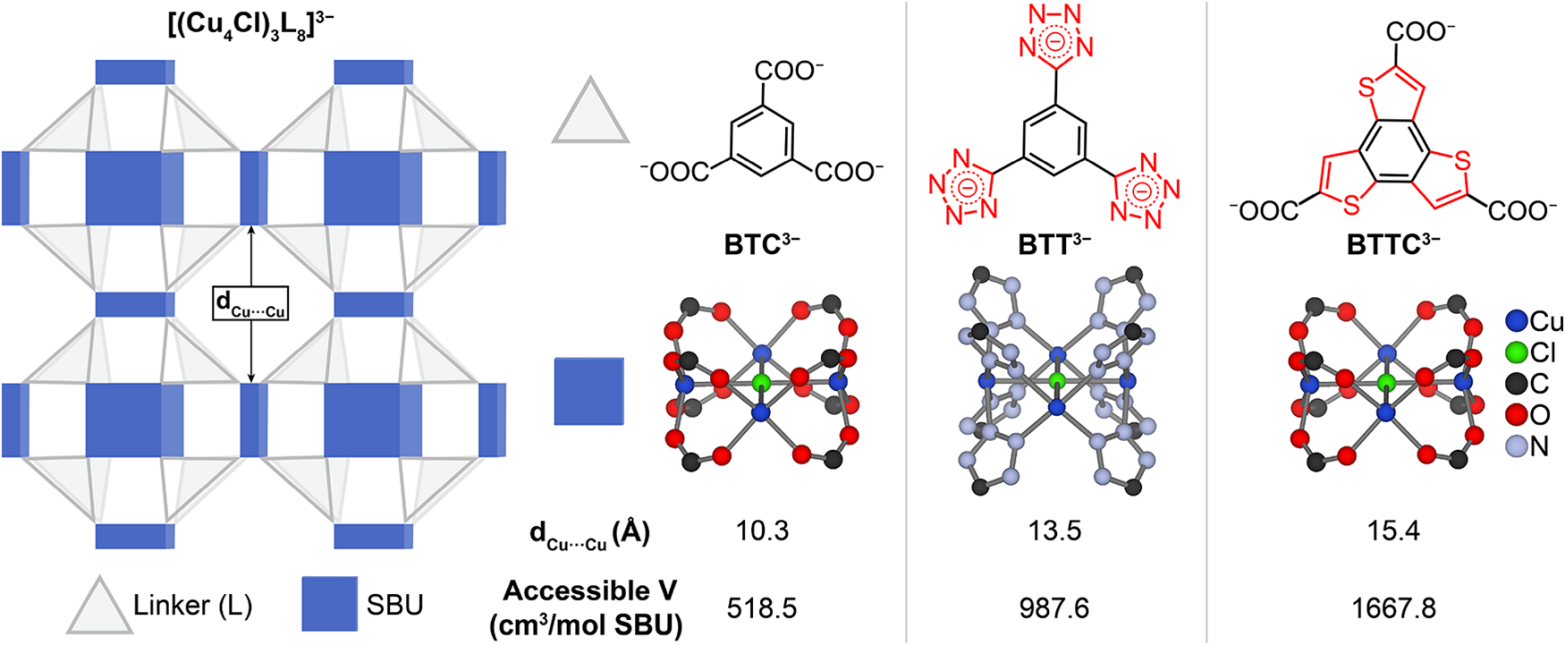
Polyhedral representation of isoreticular anionic [(Cu_4_Cl)_3_L_8_]^3–^ frameworks. The table shows the linkers, [Cu_4_Cl]^7+^ secondary building units (SBUs), crystallographic Cu···Cu distances (d_Cu···Cu_), and accessible pore volumes.

In QSSEs, Li^+^ conducts as a solvated complex, raising the question of whether confined pores impose size-selective and/or interaction-driven constraints on the solvated Li^+^ species. Narrow channels may restrict the passage of the solvated ion, whereas overly spacious pores may reduce directed transport.^19,20^ Furthermore, strong interactions between solvated Li^+^ and linker functionalities may slow ion motion.^21^ In addition, larger pore size may accommodate more conducting species, leading to higher conductivity.

To probe how pore size and linker functionality affect Li^+^ transport, we constructed the isoreticular anionic MOF series using BTC^3–^ (1,3,5-benzenetricarboxylate), BTT^3–^ (1,3,5-benzene-tris-tetrazolate), and BTTC^3–^ (benzo­[1,2-b:3,4-b':5,6-b'']­trithiophene-2,5,8-tricarboxylate) linkers (Figure 1), which systematically enlarge the pore size and introduce coordinating functionalities, such as the tetrazolate N atoms and thiophene S atoms. By combining Li^+^ conductivity measurements with ^7^Li magic-angle-spinning solid-state nuclear magnetic resonance (^7^Li MAS ssNMR), we directly correlate structural features with ion dynamics in these defined pore environments. This approach reveals how solvation, confinement, and framework functionality jointly govern Li^+^ transport, thus providing mechanistic insights on the rational design of high-performance MOF-based electrolytes.

Using methods adapted from previous reports,^16–18^ we synthesized (DMA)_3_[(Cu_4_Cl)_3_BTC_8_] ((DMA)­CuBTC), HCu­[(Cu_4_Cl)_3_BTT_8_] (HCuCuBTT), and (DMA)_3_[(Cu_4_Cl)_3_BTTC_8_] ((DMA)­CuBTTC), where DMA^+^ (dimethylammonium), H^+^, and Cu^2+^ are the extra-framework cations. We then exchanged these extra-framework cations for Li^+^ (see the Supporting Information for the complete procedures) (Figure 2A). For (DMA)­CuBTC, we exchanged the DMA^+^ for Li^+^ in a 2 M LiCl methanol solution at 85 °C for 14 days. Inductively coupled plasma-mass spectrometry (ICP-MS) confirmed stoichiometric exchange, as indicated by an experimental Li:Cu of 0.23, which closely correlates with the expected ratio of 0.25 for Li_3_[(Cu_4_Cl)_3_BTC_8_] (LiCuBTC) (Figure 2B). The Li^+^ exchange in HCuCuBTT proceeded similarly as in (DMA)­CuBTC; we achieved quantitative exchange to yield Li_3_[(Cu_4_Cl)_3_BTT_8_] (LiCuBTT) by allowing the reaction of HCuCuBTT with a 2 M LiCl solution at 60 °C for 7 days (Figure 2B). Because (DMA)­CuBTTC decomposes in the presence of LiCl, the cations in this MOF were exchanged in a 1 M LiNO_3_ *N*,*N*-dimethylformamide solution at 60–80 °C for up to 6 days (Figure 2A). After a 6-day exchange, the Li:Cu ratio is 0.43, indicating a Li^+^ incorporation of ∼175% with pores occupied by excess LiNO_3_ that cannot be easily washed out. The excess LiNO_3_ adsorption can be mitigated via shorter exchange durations of 1 day or less, leading to Li^+^ incorporation between 100% and 150% (Figure 2B). Based on this experimental evidence, we propose that the large octahedral cages could accommodate the excess Li^+^, supported by the ssNMR measurements discussed below. We describe the Li^+^-exchanged [CuBTTC]^3–^ as Li_*a*_[(Cu_4_Cl)_3_BTTC_8_]­(NO_3_)_*x*_, where *x* = *a*–3; we determined *a* using ICP-MS. For simplicity, we will refer to these materials as LiCuBTTC.

**Figure 2:**
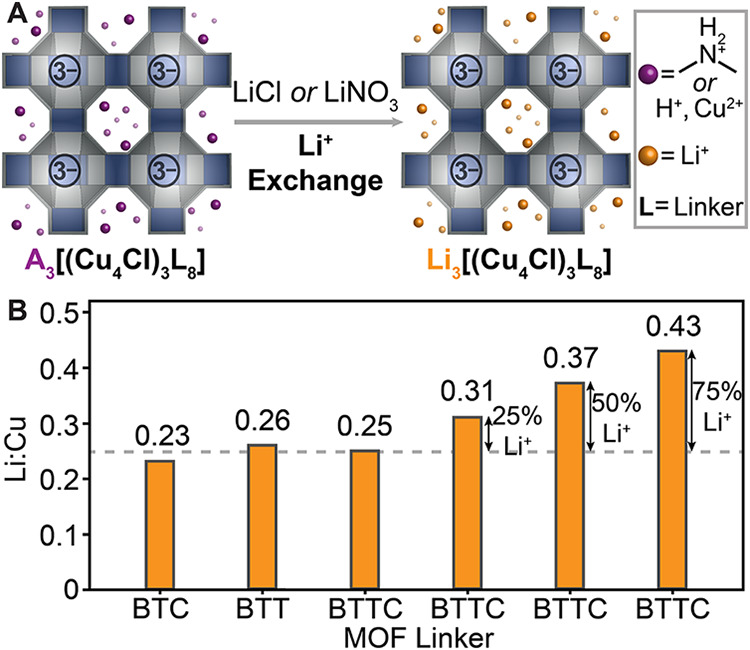
(A) Li^+^ exchange of A_3_[(Cu_4_Cl)_3_L_8_], where A^+^ and L^3–^ are the extra-framework cation and MOF linker, respectively. (B) Experimental Li:Cu ratios based on concentrations determined by ICP-MS. The gray dashed line represents the expected Li:Cu ratio of 0.25 in Li_3_[(Cu_4_Cl)_3_L_8_] after quantitative Li^+^ exchange.

To prepare the Li^+^-exchanged isoreticular MOFs for conductivity measurements, we activated the materials to remove the solvent in the pores. After grinding the activated MOFs into a powder, we added propylene carbonate (PC) to provide a solvated environment that can promote Li^+^ conduction through the pores. We normalized the amount of PC added to the accessible pore volume of each MOF. We added 5 PC per [Cu_4_Cl]^7+^ SBU to LiCuBTC, yielding LiCuBTC-PC. We used LiCuBTC as the reference and added similar amounts of PC per accessible volume for LiCuBTT and LiCuBTTC (Table S3 and Figures S1–S11). Even with the added PC, the materials remained as dry free-flowing powders, which qualifies them as QSSEs. To measure conductivity, we pelletized each MOF-PC powder, sandwiched the pellet between two stainless-steel spacers, and sealed within airtight coin cells. Variable-temperature potentiostatic electrochemical impedance spectroscopy (VT-PEIS) was measured between 25 and 75 °C (Figures S12–S14), and the Li^+^ conductivity was acquired by fitting the Nyquist plots to the appropriate equivalent circuit (see the Supporting Information for details). To obtain the activation energies, we fitted the conductivities at various temperatures with the linear Nernst–Einstein relation $\ln\left(\sigma \right)\propto -\frac{E_{a}}{k_{B}T}$ (Figure S15), where σ is the conductivity, *E*_a_ is the thermal activation energy, *k*_B_ is the Boltzmann constant, and *T* is the temperature. Powder X-ray diffraction (PXRD) confirmed that the frameworks were preserved after all treatments and electrochemical measurements (Figure S16).

Despite linker variations, we observe that the Li^+^ conductivities at 25 °C fall on the order of 10^–5^ S/cm for all three materials with quantitative Li^+^ exchange (Figure 3A and Table S4; LiCuBTC-PC: 2.6 × 10^–5^ S/cm, LiCuBTT-PC: 1.1 × 10^–5^ S/cm, LiCuBTTC (100% Li^+^)-PC: 2.6 × 10^–5^ S/cm). Two structural factors guide the interpretation of these results: (1) pore-limiting diameters/accessible pore volumes and (2) linker functionalities (Figure 1). The pore-limiting diameter determines whether Li^+^ needs to undergo partial desolvation to move through the narrowest path, whereas the accessible pore volume dictates whether Li^+^ can maintain a stabilizing solvation shell. Both parameters increase from LiCuBTC to LiCuBTT and LiCuBTTC (Figure 1). Previous work showed that ion conductivity decreases for small-pore materials due to congestion at the pore windows, with conductivity increasing as pore volume grows and eventually plateauing as the system approaches bulk-liquid limit.^11^ Given the Li^+^ conductivities across the three systems fall on the same order of magnitude (Figure 3A), we propose that their pore sizes are sufficiently large to support solvent-assisted, liquid-like Li^+^ transport.

**Figure 3:**
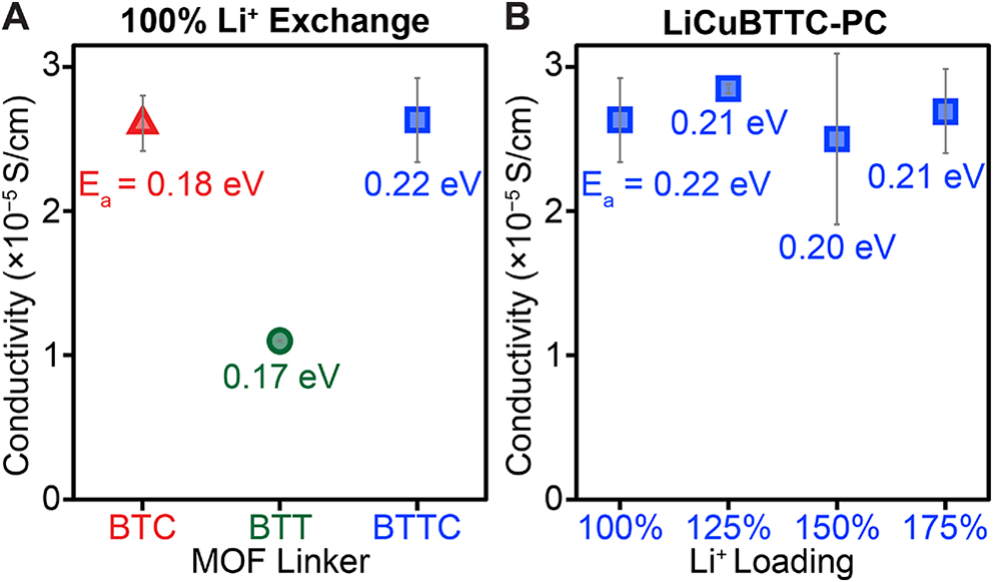
Li^+^ conductivities at 25 °C of (A) isoreticular MOFs with quantitative Li^+^ exchange and (B) LiCuBTTC with different Li^+^ loadings. The thermal activation energies (*E*_a_) are shown.

To explore the role of chemical functionality on Li^+^ conductivity, we rely on the tetrazolate N atoms in LiCuBTT and thiophene S atoms in LiCuBTTC. Although these groups present potential lithium binding sites that could modulate Li^+^ transport relative to LiCuBTC-PC, our results indicate that their influence is secondary to accessible pore volume (Figure 1), which largely governs the liquid-like Li^+^ conduction. All three isoreticular MOFs exhibit conductivities on the order of 10^–5^ S/cm; however, LiCuBTT-PC displays a lower conductivity of 1.1 × 10^–5^ S/cm, approximately half that of LiCuBTC-PC and LiCuBTTC-PC (2.6 × 10^–5^ S/cm). The pore size of LiCuBTT-PC is intermediate between those of LiCuBTC-PC and LiCuBTTC-PC (Figure 1), while the latter two exhibit identical conductivity at 25 °C despite their different pore dimensions (Figure 3A). This comparison indicates that pore size alone does not dictate Li^+^ transport in this system and instead highlights the role of linker functionality. We therefore attribute the lower conductivity in LiCuBTT-PC primarily to differences in linker functionality, suggesting Li^+^–tetrazolate N interactions consistent with prior studies.^22,23^ In addition to probing the Li^+^ transport in materials with quantitative Li^+^ exchange (Figure 3A), we examined the effect of different Li^+^ loadings on conductivity in LiCuBTTC-PC; we observe similar conductivities (2.5–2.8 × 10^–5^ S/cm at 25 °C) for Li^+^ incorporation ranging between 100% and 175% (Figure 3B). We will explain these conductivity trends from a molecular perspective using ^7^Li ssNMR below. From the VT-PEIS, we obtain thermal activation energies of ∼0.2 eV across the isoreticular series (Figures 3 and S15). These low activation energies suggest that the anionic charge in the [(Cu_4_Cl)_3_L_8_]^3–^ framework is well-delocalized, enabling efficient Li^+^ migration — a key feature of high-performing QSSEs.

To understand Li^+^ transport in the pores of the anionic MOFs at the molecular level, we studied the lithium environments and dynamics using ^7^Li MAS ssNMR (Figure 4). We investigated the role of PC solvation on Li^+^ transport by probing the ^7^Li species in activated LiCuBTC and LiCuBTC-PC (Figure 4A). In LiCuBTC, two major chemical environments appear at 0.4 ppm and −39.6 ppm. As the ^7^Li chemical shift is modestly sensitive to its environment in diamagnetic systems,^24–26^ we attribute the −39.6 ppm resonance to a Li^+^ species that is close to the paramagnetic Cu^2+^ resulting in the large negative shift.^27^ To deduce the Li^+^ mobility and thus its transport behavior, we look at the intensity of spinning sidebands and peak width.^28–30^ Smaller spinning sidebands and narrower peaks indicate faster tumbling and therefore more liquid-like motion.^31^ The pronounced spinning sidebands and broad signals in LiCuBTC reveal strong interactions between Li^+^ and the [CuBTC]^3–^ framework (Figure 4A). This corresponds to a solid-like Li^+^ with low mobility, consistent with a Li^+^ conductivity ≪ 10^–9^ S/cm. Upon PC addition to form LiCuBTC-PC, two major chemical environments remain but they exhibit new chemical shifts at 1.4 ppm and −9.4 ppm with significantly reduced spinning sidebands (Figure 4A). The attenuation of sidebands indicates enhanced Li^+^ mobility, resulting in liquid-like transport in LiCuBTC-PC. We note that the sidebands of the −9.4 ppm signal almost completely disappear while the peak sharpens, implying its contribution as the dominant conducting species. Based on the experimental evidence, we suggest the following physical picture of where the two ^7^Li species locate in the MOF (Figure 4A inset). The signal at the higher frequency likely corresponds to the Li^+^ confined within the octahedral cage (green area in Figure 4A inset), contributing minimally to conductivity. While the signal at the lower frequency represents the Li^+^ in the open channels (purple area in Figure 4A inset), which support long-range translocation leading to major contribution in Li^+^ transport. Since the signal in purple at −9.4 ppm in LiCuBTC-PC is paramagnetically shifted, we speculate that the Li^+^ is proximal to the solvent-accessible axial binding sites of Cu^2+^ ions that decorate the pore-limiting window in the channels (Figure 4A inset).

**Figure 4:**
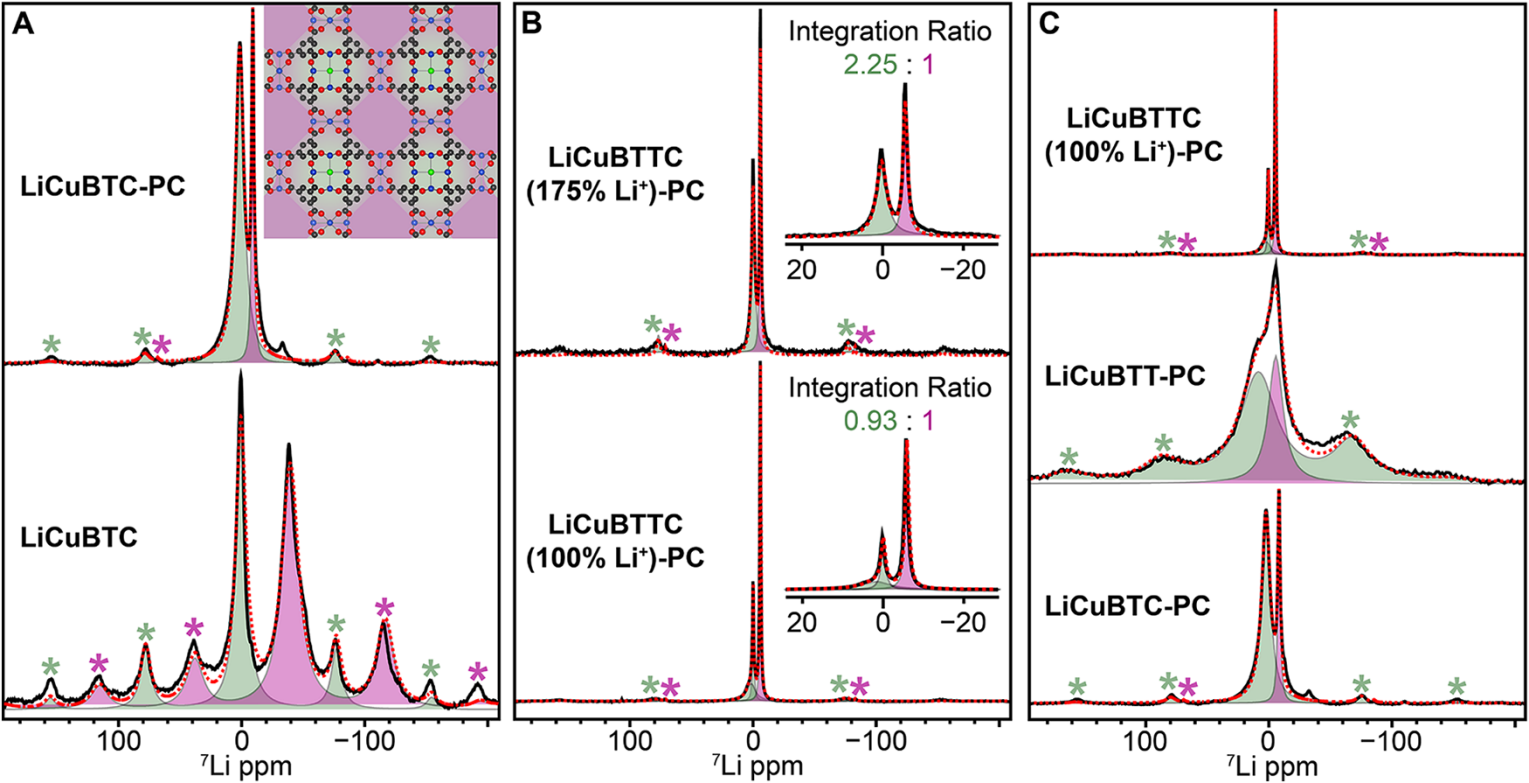
^7^Li ssNMR spectra collected at 12 kHz magic-angle-spinning rate for (A) LiCuBTC and LiCuBTC-PC; (B) LiCuBTTC (100% Li^+^)-PC and LiCuBTTC (175% Li^+^)-PC; (C) LiCuBTC-PC, LiCuBTT-PC, and LiCuBTTC (100% Li^+^)-PC. The solid black and dashed red lines indicate the experimental and fitted spectra, respectively. The shaded areas under peaks represent the fits of individual peaks, and their spinning sidebands are indicated with asterisks. Chemical shifts are referenced to 1 M LiCl in D_2_O as 0 ppm. The inset in (A) shows the proposed environments for the two signals, where the green and purple resonances represent Li^+^ in octahedral cages and channels, respectively.

^7^Li ssNMR further explains the similar conductivities in LiCuBTTC-PC with different Li^+^ loadings (Figures 3B and 4B). Going from 100% to 175% Li^+^, the integration of the ∼0 ppm signal (green) increases, whereas that of the ∼−6 ppm signal (purple) remains unchanged. Consequently, the integration ratio increases from 0.93 to 2.25 (Figure 4B), consistent with a ∼70% increase in total Li^+^ amount that matches the ICP-MS results (Figure 2B). The larger integration of the signal in green in the 175% Li^+^ material arises from the increase of a paramagnetism-influenced ^7^Li species (T_1_ = 0.02 s) and the emergence of a species in a diamagnetic environment (T_1_ = 1.95 s) (Table S5). Since a higher amount of species corresponding to the green signal does not affect conductivity (Figure 3B), it reveals that the species corresponding to the purple signal at ∼−6 ppm is the major contributor that governs Li^+^ transport (Figure 4B). Given this, the similar lower-frequency resonances in LiCuBTC-PC (−9.4 ppm) and LiCuBTT-PC (−6.3 ppm) imply their assignment as the dominant conducting species residing in the open pore channels (Figure 4C). As both the 100% and 175% Li^+^ LiCuBTTC-PC contain 0.5 of the more mobile Li^+^ per SBU, we speculate the origin of the major conducting species is structural in nature rather than dictated by Li^+^ loadings.

Using ^7^Li ssNMR, we elucidated the origin of comparable Li^+^ conductivities in the isoreticular series (Figures 3A and 4C). As the intensities of spinning sidebands significantly decrease upon PC addition (Figures 4A and S17–S19), Li^+^ transport becomes mobile and liquid-like. Given the similar chemical shifts of the major conducting species (−6.2 to −9.4 ppm; purple signals in Figure 4C), we infer they reside in similar environments within the PC-filled channels despite linker variations (purple area in Figure 4A inset). The liquid-like transport and similar local environments of major conducting species yield comparable Li^+^ transport properties across the isoreticular series. Although on the same order of magnitude, LiCuBTT-PC shows a conductivity that is approximately two-fold lower than those of LiCuBTC-PC and LiCuBTTC-PC (Figure 3A). We attribute this to a greater solid-like character, as LiCuBTT-PC shows the most prominent spinning sidebands and broadest signals among the three materials (Figure 4C). This may arise from the interactions between the Li^+^ and the tetrazolate nitrogens in BTT^3–^ linker, restricting ion motion.^22^ Since the BTT^3–^–Li^+^ interactions predominantly reduce the mobility of the minor conducting species (green), while exerting little influence on the major conducting species (purple), the overall conductivity of LiCuBTT-PC is modestly lower relative to the other two materials. While the spinning sideband analysis offers a reliable qualitative indicator of Li^+^ mobility, we complement it with a semiquantitative analysis based on variable-temperature ^7^Li T_1_ measurements (temperature-dependent T_1_ trends shown in Figure S20), whose trends across the MOF series are fully consistent with the sideband analysis and the measured Li^+^ conductivities.

In conclusion, we have elucidated the molecular factors governing Li^+^ transport in MOF-based QSSEs using an isoreticular series of Li_3_[(Cu_4_Cl)_3_L_8_] MOFs. The liquid-like transport and similar local environments of the major conducting species result in comparable conductivities across the series. Within this pore size regime, Li^+^ transport is largely independent of the pore dimensions, while the chemical functionality can modulate conductivity. Our study offers molecular-level insights on Li^+^ conduction in MOF-based electrolytes, thus establishing the design principles for developing efficient QSSEs.

## Experimental Methods

### General Materials and Methods

Detailed synthetic procedures and compositional analyses are provided in the Supporting Information. The isoreticular LiCuBTC, LiCuBTT, and LiCuBTTC were prepared through postsynthetic Li^+^ exchange of their corresponding parent frameworks of (DMA)­CuBTC, HCuCuBTT, and (DMA)­CuBTTC, respectively. The parent frameworks were synthesized according to modified literature procedures.^16–18^ PXRD patterns were collected using a Bruker D8 Advance II diffractometer with Cu Kα radiation to verify framework crystallinity. The extent of Li^+^ incorporation was determined by ICP-MS.

### Preparation of QSSEs

QSSEs were prepared by grinding activated Li^+^-exchanged MOFs with battery-grade PC. The amount of PC incorporated into each material was quantified by solution ^1^H NMR on the digested samples. The resulting QSSEs were pelletized and assembled into airtight coin cells for ion conductivity measurements.

### Li^+^ Conductivity Measurements

Li^+^ conductivities of LiCuBTC-PC, LiCuBTT-PC, and LiCuBTTC-PC were measured by VT-PEIS using BioLogic VSP potentiostat over a frequency range of 500 kHz to 100 Hz with a 50 mV perturbation amplitude. Conductivities were probed between 25 and 75 °C. Nyquist plots were fitted using an equivalent circuit model to extract bulk resistances and calculate Li^+^ conductivities. Additional experimental details, data fitting, and analyses are provided in the Supporting Information.

### Probing Li^+^ Dynamics

We probed Li^+^ dynamics using ^7^Li MAS ssNMR on Bruker Ascend 400 MHz spectrometer with a 3.2 mm HX double-channel probe. Spectra were acquired with a MAS rate of 12 kHz and referenced to 1 M LiCl in D_2_O as 0 ppm. T_1_ relaxation times were measured using a saturation recovery pulse sequence. Both quadrupolar interaction and chemical shift anisotropy were included in the spectral fitting. Detailed information is available in the Supporting Information.

## Supplementary Material



## Notes

Materials; experimental methods; and characterization, including NMR, PEIS, PXRD; and ICP-MS (PDF)
