# Supplementary material for: Molecular Insights Shaping the Design of Metal–Organic Framework-Based Electrolytes
Source: Chem Mater. 2026 Jun 19;38(13):6956–61. doi: 10.1021/acs.chemmater.6c01203 (PMC13373998; doi:10.1021/acs.chemmater.6c01203)
Supplement: Supplementary file 1 [file cm6c01203_si_001.pdf]

Supporting Information for

Molecular Insights Shaping the Design of Metal-Organic Framework-based Electrolytes

Shoushou He,[1] Julius J. Oppenheim,[1] Keiichiro Maegawa,[1] Zhentao Yang,[1] Yunyao Xu,[2] Ann E. McDermott,[2] Mircea Dincă*[1]

[1] *Department of Chemistry, Princeton University, Princeton, NJ 08540, USA*
[2] *Department of Chemistry, Columbia University, New York, NY 10027, USA*

Table of Contents

Synthetic Details *3*

Table S1 4

Table S2 5

Characterization Data *6*

Table S3 6

Table S4 6

Table S5 6

Figure S1 7

Figure S2 8

Figure S3 8

Figure S4 9

Figure S5 9

Figure S6 10

Figure S7 10

Figure S8 11

Figure S9 11

Figure S10 12

Figure S11 12

Figure S12 13

Figure S13 13

Figure S14 14

Figure S15 15

Figure S16 15

Figure S17 16

Figure S18 16

Figure S19 17

Figure S20 18

References *19*

Synthetic Details

General Information

$CuCl_2 \cdot 2H_2O$ was purchased from Alfa Aesar. 1,3,5-benzenetricarboxylic acid and all solvents were purchased from Sigma Aldrich. 1,3,5-benzene-tris-tetrazole and benzo[1,2-b:3,4-b':5,6-b'']trithiophene-2,5,8-tricarboxylic acid were purchased from ET Co., Ltd. and Ambeed, respectively. Reactions under inert atmosphere were carried out using standard Schlenk line and glovebox techniques. Dry and deoxygenated solvents were prepared by elution through a dual-column solvent purification system.

$[(CH_3)_2NH_2]_3[(Cu_4Cl)_3(BTC)_8]$ ((DMA)CuBTC)

The synthesis for $[(CH_3)_2NH_2]_3[(Cu_4Cl)_3(BTC)_8]$ was adapted from a previously reported protocol.[1] $CuCl_2 \cdot 2H_2O$ (13.6 g, 1 mmol, 2 equiv.) and 1,3,5-benzenetricarboxylic acid (8.4 g, 0.5 mmol, 1 equiv.) were dissolved in a mixture of *N,N*-dimethylacetamide (400 mL) and water (8 mL). Reaction was heated at 100 °C for 2 days. The crystals were washed with 2× ~30 mL *N,N*-dimethylformamide (DMF), followed by 3× ~30 mL methanol. The crystals were dried *in vacuo* at room temperature to afford turquoise crystals.

$HCu[(Cu_4Cl)_3(BTT)_8]$ (HCuCuBTT)

The synthesis for $HCu[(Cu_4Cl)_3(BTT)_8]$ was adapted from a previously reported protocol.[2] In a vial, $CuCl_2 \cdot 2H_2O$ (201 mg, 1.18 mmol, 8.6 equiv.) was dissolved in methanol (8 mL). In another vial, 1,3,5-benzene-tris-tetrazole (40 mg, 0.138 mmol, 1 equiv.) was dissolved in DMF (8 mL). The two solutions were combined and 1 M HCl(aq) was added to acidify to pH ~1. The reaction was let sit at room temperature for 3 days to yield $HCu[(Cu_4Cl)_3(BTT)_8]$ as a green powder, which was washed with 2× ~30 mL DMF, followed by 1× ~30 mL methanol. The powder was dried *in vacuo* at room temperature to afford a dark blue powder, which was stored in a N_2-filled glovebox for its air-sensitivity.

$[(CH_3)_2NH_2]_3[(Cu_4Cl)_3(BTTC)_8]$ ((DMA)CuBTTC)

The synthesis for $[(CH_3)_2NH_2]_3[(Cu_4Cl)_3(BTTC)_8]$ was adapted from a previously reported protocol.[3] In a vial, $CuCl_2 \cdot 2H_2O$ (682 mg, 4 mmol, 2 equiv.), benzo[1,2-b:3,4-b':5,6-b'']trithiophene-2,5,8-tricarboxylic acid (757 mg, 2 mmol, 1 equiv.), *N,N*-dimethylacetamide (20 mL), and water (0.8 mL) were combined. The reaction was heated at 100 °C for 3 days to yield light green crystals, which were washed with 2× ~40 mL DMF, followed by 1× ~40 mL tetrahydrofuran (THF). The crystals were dried *in vacuo* at room temperature.

$Li_3[(Cu_4Cl)_3(BTC)_8]$ (LiCuBTC)

$[(CH_3)_2NH_2]_3[(Cu_4Cl)_3(BTC)_8]$ (250 mg) and 2 M LiCl methanol solution (25 mL) were loaded in a pressure tube. The reaction was heated at 85 °C for 14 days. Fresh salt solution was exchanged daily. The crystals were washed with 4× ~20 mL methanol and dried *in vacuo* at room temperature.

$Li_3[(Cu_4Cl)_3(BTT)_8]$ (LiCuBTT)

In a N_2-filled glovebox, $HCu[(Cu_4Cl)_3(BTT)_8]$ (90 mg) and 2 M LiCl methanol solution (40 mL) were loaded in a Schlenk tube. The reaction was heated at 60 °C with 300 rpm stirring for 7 days. The salt solution was exchanged daily. The solid was washed with 4× ~30 mL methanol and dried *in vacuo* at room temperature.

$Li_a[(Cu_4Cl)_3BTTC_8](NO_3)_x$ (LiCuBTTC), where $x = a - 3$

1 M $LiNO_3$ DMF solution (20 mL) was added to $[(CH_3)_2NH_2]_3[(Cu_4Cl)_3(BTTC)_8]$ (20 mg). The reaction conditions for different amounts of Li^+ incorporation are shown in the table below. After the salt solution was removed, the light green crystals were washed with 3× ~40 mL DMF, followed by 1× ~40 mL THF. The crystals were dried *in vacuo* at room temperature.

Table S1. Reaction conditions for $Li_a[(Cu_4Cl)_3BTTC_8](NO_3)_x$ (LiCuBTTC), where $x = a - 3$.

**%Li^+ relative to $Li_3[(Cu_4Cl)_3BTTC_8]$**	***a***	***x***	**Temperature (°C)**	**Time**
100%	3.0	0	60	15 min
125%	3.7	0.7		100 min
150%	4.5	1.5		20 hr
175%	5.2	2.2	80	6 days

Instrumentation and Measurements

Powder X-ray Diffraction (PXRD)

PXRD patterns were collected on Bruker D8 Advance II diffractometer with a Ni-filtered Cu Kα radiation. The tube voltage and current were set to 40 kV and 40 mA, respectively.

Inductively Coupled Plasma-Mass Spectrometry (ICP-MS)

Each sample (~1–3 mg) was digested in OmniTrace Ultra Nitric acid (2 mL); the solution was first diluted by 10-fold with MilliQ H_2O to yield Solution A, which was then diluted with 2% HNO_3 for an overall 200-fold dilution to yield Solution B. We quantified [Cu] and [Li] in Solution B using Agilent 7900 ICP-MS to determine the extent of Li^+ exchange.

^7Li Magic-Angle Spinning Solid-State NMR (MAS ssNMR)

The samples were packed into a 3.2 mm ZrO_2 rotor with a VESPEL drive cap in a Argon-filled glovebox. We performed the ^7Li ssNMR on Bruker Ascend™ 400 MHz spectrometer at the Princeton Chemistry NMR Facility. A 3.2 mm HX double-channel probe was tuned to the ^7Li frequency. Proton decoupling was tested to show that it is not necessary for spectral acquisition due to the paramagnetism from Cu^{2+}. The typical 90° pulse length for ^7Li at 82 W was 3 μs. ^7Li chemical shifts were referenced to 1 M LiCl in D_2O as 0 ppm. ^7Li spectra were acquired at 298 K with MAS rate of 12 kHz.

Spectra were processed and fitted with Fit Solids NMR Models (sola) in Topspin 4.5.0. Both Quadrupolar interaction and chemical shift anisotropy were included in the spectral fitting. The signals were fitted to determine their chemical shifts of different environments and their integrations. In order to quantitatively measure the population of different species, we set the pulse delay time to at least 3.5–5 times of T_1 to ensure full recovery to thermal equilibrium; we summarize the pulse delay times in the table below. We measured T_1 relaxation times with a saturation recovery pulse sequence; the results are shown in **Table S2**. We note that for LiCuBTTC (175% Li^+) and LiCuBTTC (175% Li^+)-PC, the peak at ~0 ppm displays two T_1 relaxation times despite the same chemical shift.

Table S2. Pulse delay times used for samples.

Sample	Pulse delay (s)
LiCuBTC	0.5
LiCuBTC-PC	0.8
LiCuBTT	0.5
LiCuBTT-PC	0.5
LiCuBTTC (100% Li^+)	2
LiCuBTTC (100% Li^+)-PC	0.5
LiCuBTTC (175% Li^+)	10
LiCuBTTC (175% Li^+)-PC	7

Variable-Temperature Potentiostatic Electrochemical Impedance Spectroscopy (VT-PEIS)

Sample Preparation and Coin Cell Fabrication

We ground the MOF crystals (used 50–100 mg for each coin cell) into a fine powder, to which we added battery-grade propylene carbonate (PC) yielding MOF-PC. See **Table S3** for volume of PC added for each MOF. We pelletized each MOF-PC, sandwiched the pellet between two stainless-steel spacers, and sealed them in an airtight coin cell.

VT-PEIS Measurements

PEIS was measured using a two-probe method on the BioLogic five-channel VSP potentiostat. With an input voltage amplitude of 50 mV, PEIS was collected between a frequency range of 500 kHz–100 Hz. The coin cell was placed in the Yamato DVS402 programmable oven to measure PEIS at temperatures between 25 °C and 75 °C at a 10 °C increment.

Fitting of PEIS Data

All PEIS traces were fitted to the R1+(R2/Q1)+Q2 equivalent circuit below. In our system, R1 is zero and R2 is the resistance of the MOF-PC.

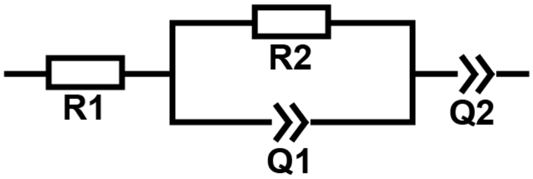

^{1}H and ^{13}C NMR

Sulfuric acid-d_2 (50 μL) was added to MOF-PC (~1 mg) and sonicated to obtain a uniform suspension, to which dimethyl sulfoxide-d_6 (600 μL) was added to yield a clear solution for NMR. Standard ^{1}H (32 scans) and ^{13}C NMR (2048 scans) spectra were collected on Neo500 and Neo402 magnetometers. We determined the amount of PC in the quasi-solid-state electrolytes by integrating its $–CH_3$ signal at ~1.23 ppm in ^{1}H NMR and referencing it to the integration of ligand signal.

Characterization Data

Table S3. Number of propylene carbonate (PC) added per $[Cu_4Cl]^{7+}$ SBU (determined by ^{1}H NMR) for electrochemical measurements.

**Materials**	**Number of PC per $[Cu_4Cl]^{7+}$ SBU**	**Volume of PC added per 50 mg MOF (μL)**
LiCuBTC-PC	5.1	17.5
LiCuBTT-PC	9.8	26.8
LiCuBTTC (100% Li^+)-PC	13.3	42.0
LiCuBTTC (125% Li^+)-PC	15.0	41.7
LiCuBTTC (150% Li^+)-PC	12.5	36.4
LiCuBTTC (175% Li^+)-PC	23.0	45.0

Table S4. Average Li^+ conductivities and thermal activation energies.

Materials	Conductivity at 25 °C (×10^{-5} S/cm)	E_a (eV)
LiCuBTC-PC	2.6 ± 0.2	0.185 ± 0.007
LiCuBTT-PC	1.11 ± 0.01	0.17 ± 0.01
LiCuBTTC (100% Li^+)-PC	2.6 ± 0.3	0.22 ± 0.01
LiCuBTTC (125% Li^+)-PC	2.85 ± 0.04	0.21 ± 0.01
LiCuBTTC (150% Li^+)-PC	2.5 ± 0.6	0.20 ± 0.02
LiCuBTTC (175% Li^+)-PC	2.7 ± 0.3	0.21 ± 0.01

Table S5. Summary of ^{7}Li ssNMR results of LiCuBTC, LiCuBTT, and LiCuBTTC with 12 kHz magic-angle spinning rate.

	**Chemical Shift (ppm)**			**Integration Ratio of Peak 1/Peak 2 or (Peaks 1+2)/Peak 3**	**T_1 Relaxation Time (s)**
	Peak 1	Peak 2	Peak 3		
**LiCuBTC**	0.4	−39.6	-	0.65	0.022 (peak 1)<br>0.010 (peak 2)
**LiCuBTC-PC**	1.4	−9.4	-	3.77	0.014 (peak 1)<br>0.024 (peak 2)

**LiCuBTT**	11.1	-	-	-	0.001
**LiCuBTT-PC**	10.8	−6.3	-	3.57	0.002 (peak 1)<br>0.001 (peak 2)
**LiCuBTTC (100% Li^+)**	4.5	0.1	−5.9	1.58	0.013 (peak 1)<br>0.038 (peak 3)
**LiCuBTTC (100% Li^+)-PC**	1.5	−0.3	−6.2	0.93	0.041 (peak 1)<br>0.070 (peak 3)
**LiCuBTTC (175% Li^+)**	−0.6	−6.0	-	2.81	0.099‡ (peak 1)<br>3.694‡ (peak 1)<br>0.030 (peak 2)
**LiCuBTTC (175% Li^+)-PC**	−0.4	−6.3	-	2.25	0.021‡ (peak 1)<br>1.955‡ (peak 1)<br>0.054 (peak 2)

‡ Peak 1 in LiCuBTTC (175% Li^+) and in LiCuBTTC (175% Li^+)-PC contains two species, which are associated with their distinct T_1 relaxation times.

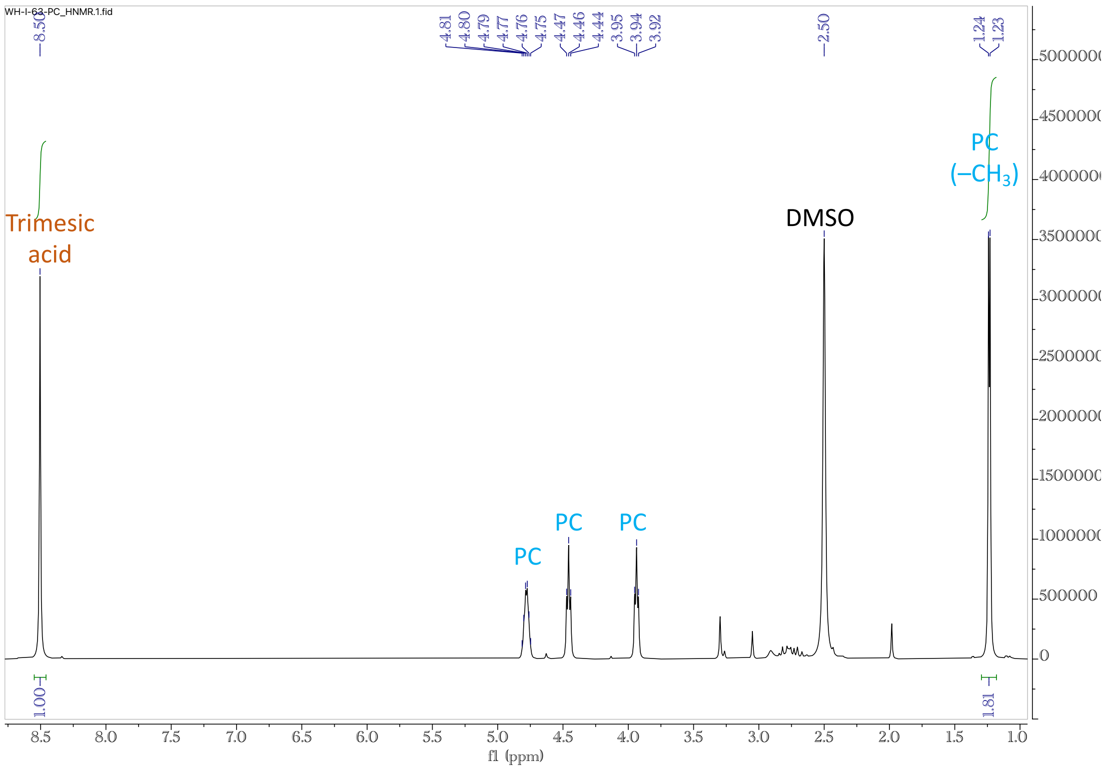

Figure S1. ^{1}H NMR of digested LiCuBTC-PC in dimethyl sulfoxide-d_6.

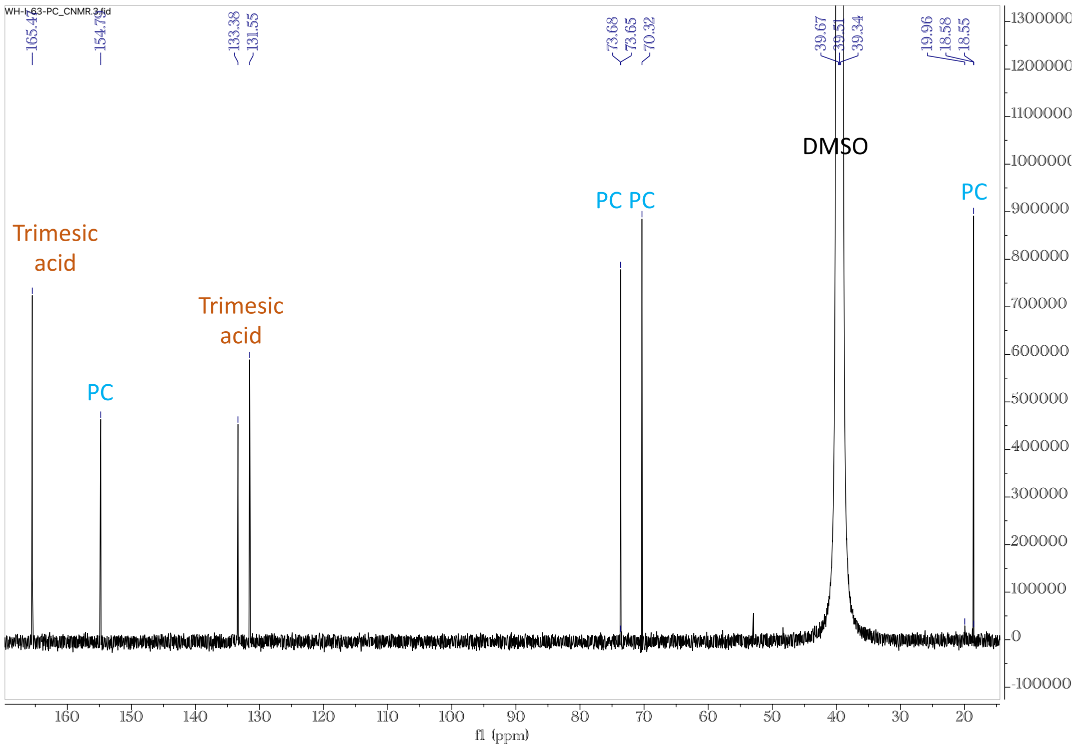

Figure S2. ^{13}C NMR of digested LiCuBTC-PC in dimethyl sulfoxide-d_6.

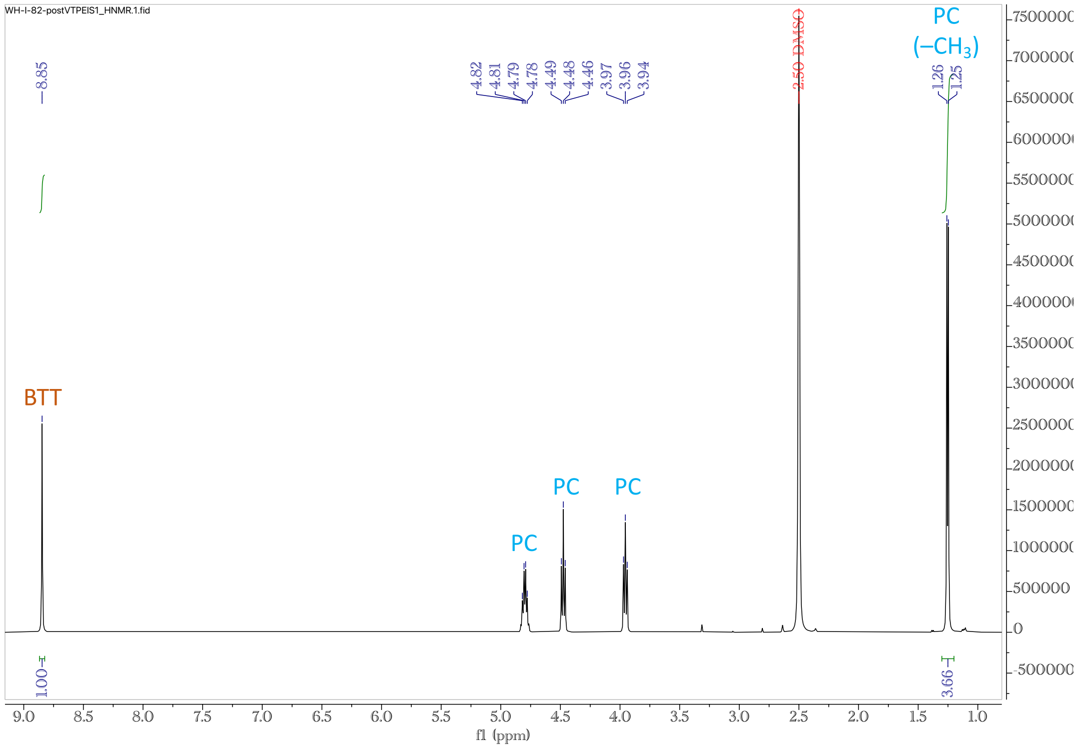

Figure S3. ^{1}H NMR of digested LiCuBTT-PC in dimethyl sulfoxide-d_6.

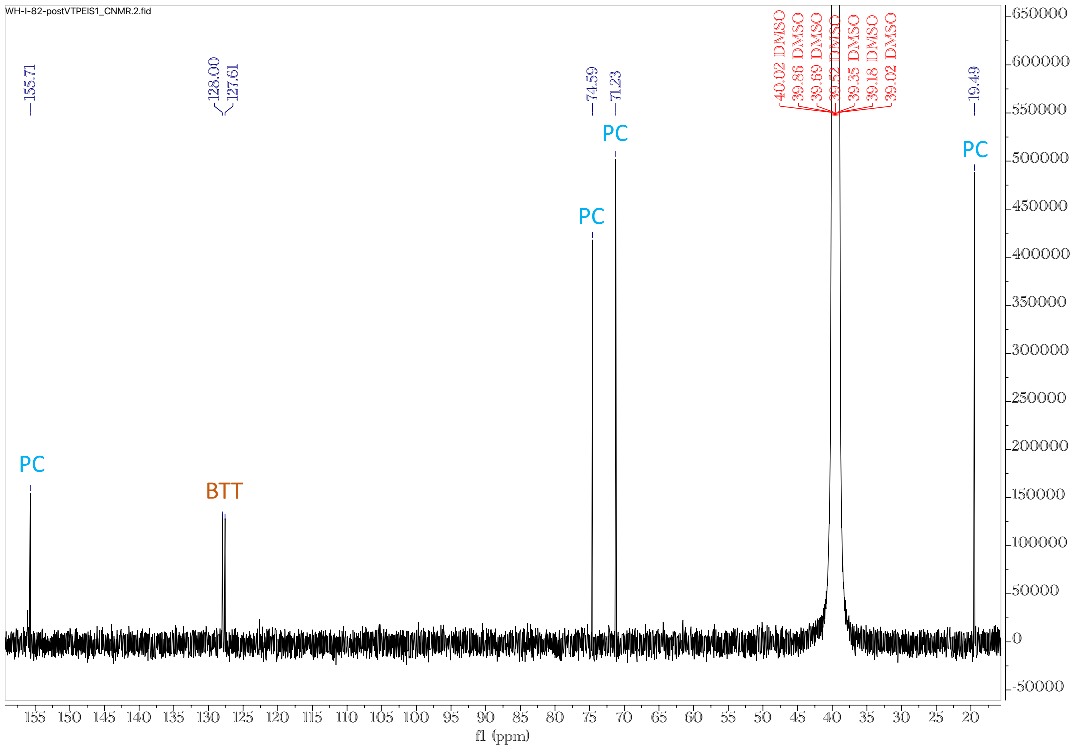

Figure S4. ^{13}C NMR of digested LiCuBTT-PC in dimethyl sulfoxide-d_6.

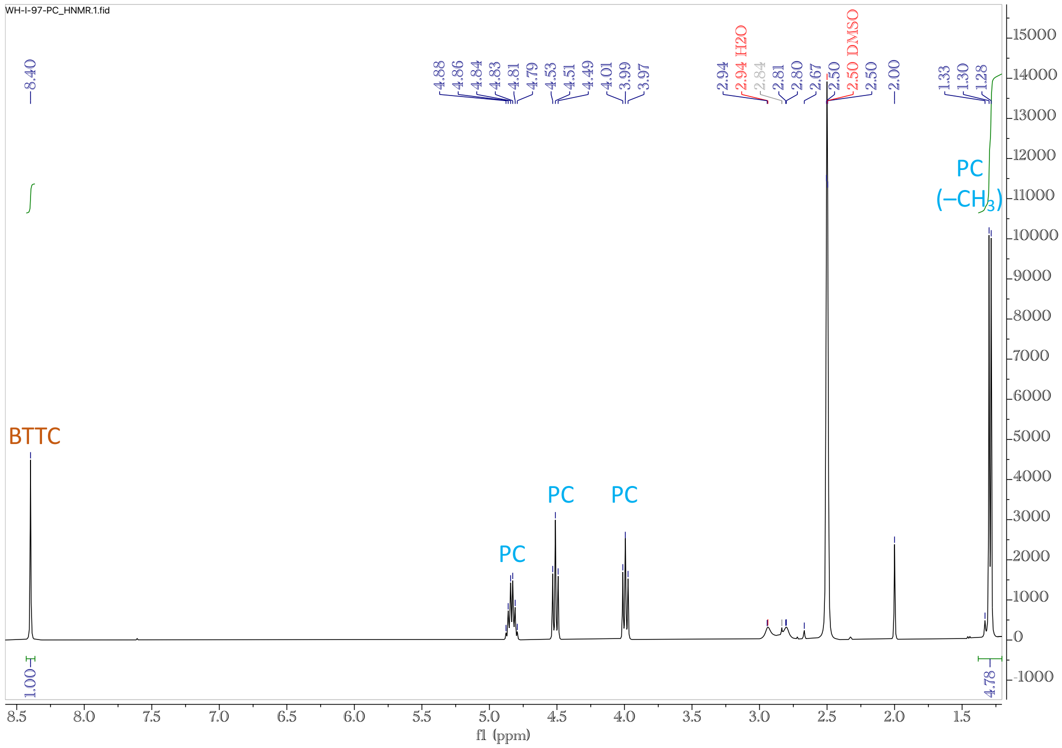

Figure S5. ^{1}H NMR of digested LiCuBTTC (100% Li^+)-PC in dimethyl sulfoxide-d_6.

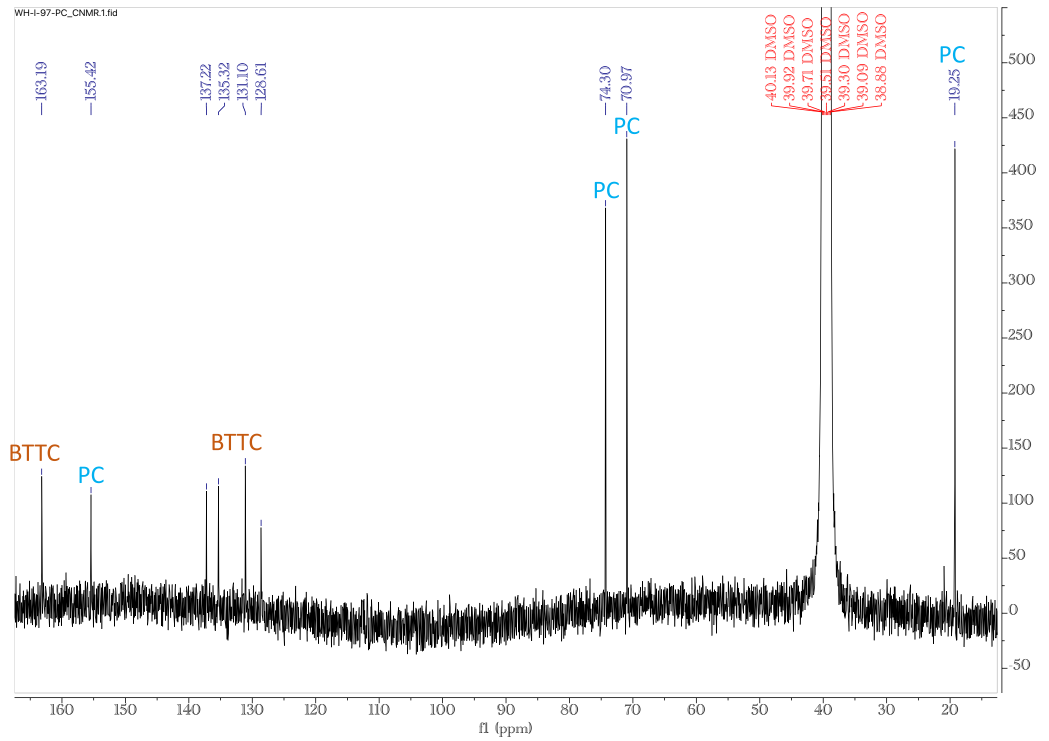

Figure S6. ^{13}C NMR of digested LiCuBTTC (100% Li^+)-PC in dimethyl sulfoxide-d_6.

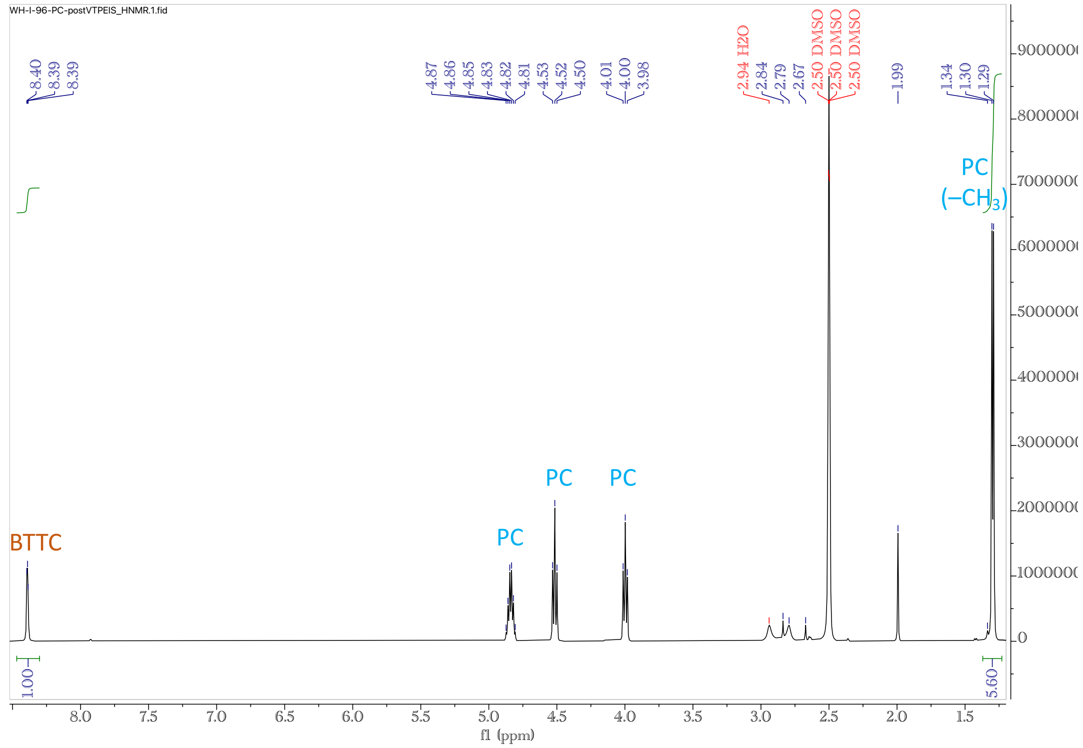

Figure S7. ^1H NMR of digested LiCuBTTC (125% Li^+)-PC in dimethyl sulfoxide-d_6.

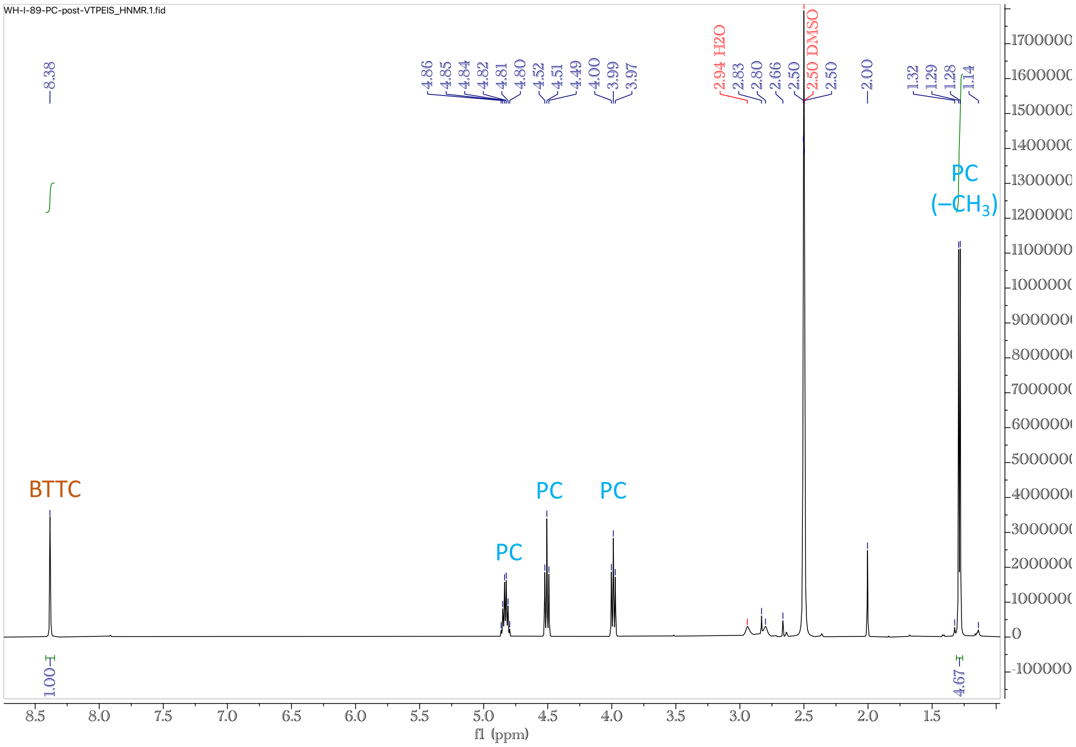

Figure S8. ^{1}H NMR of digested LiCuBTTC (150% Li^{+})-PC in dimethyl sulfoxide-d_6.

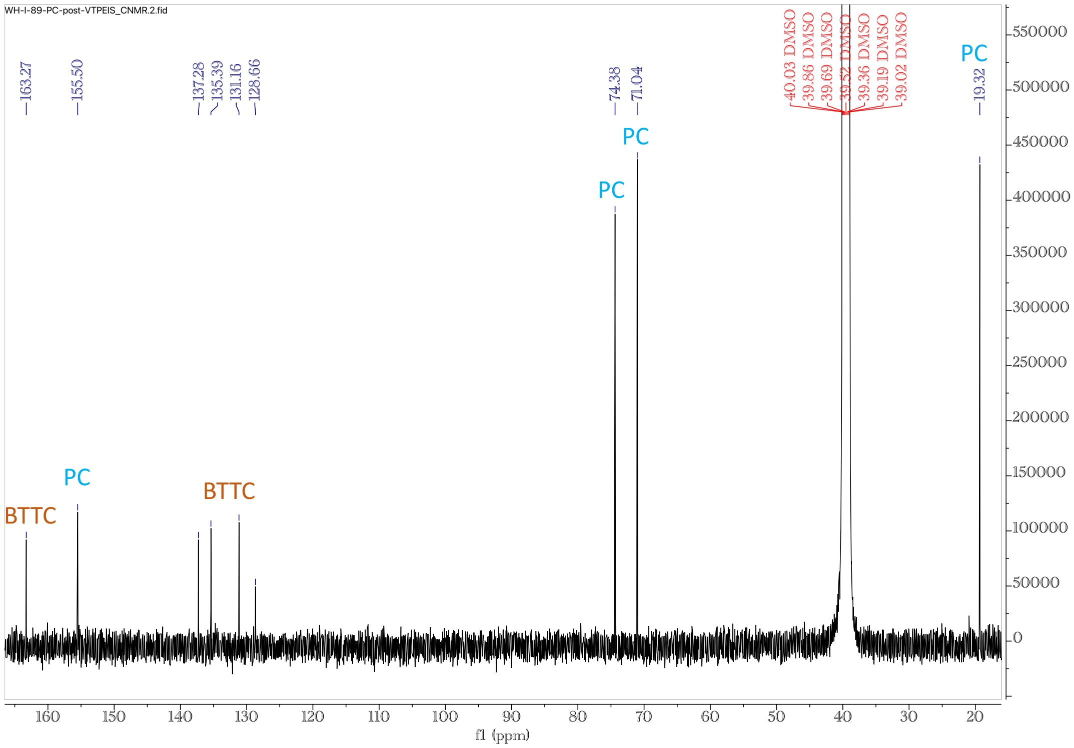

Figure S9. ^{13}C NMR of digested LiCuBTTC (150% Li^{+})-PC in dimethyl sulfoxide-d_6.

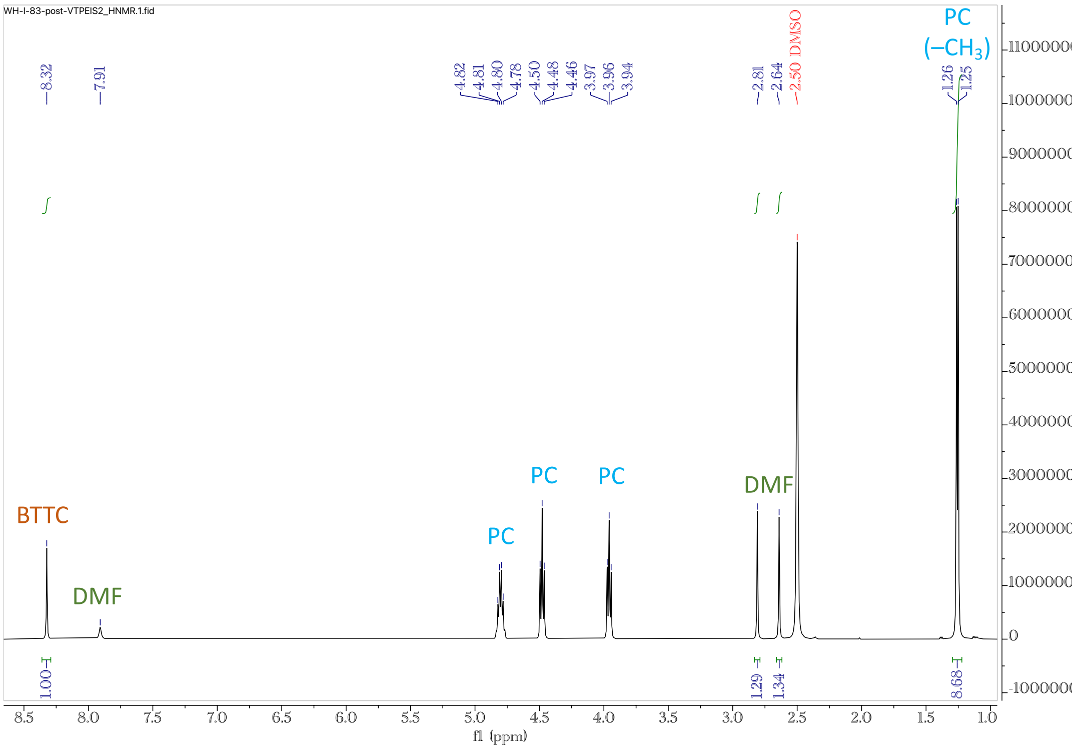

Figure S10. ^{1}H NMR of digested LiCuBTTC (175% Li^{+})-PC in dimethyl sulfoxide-d_6.

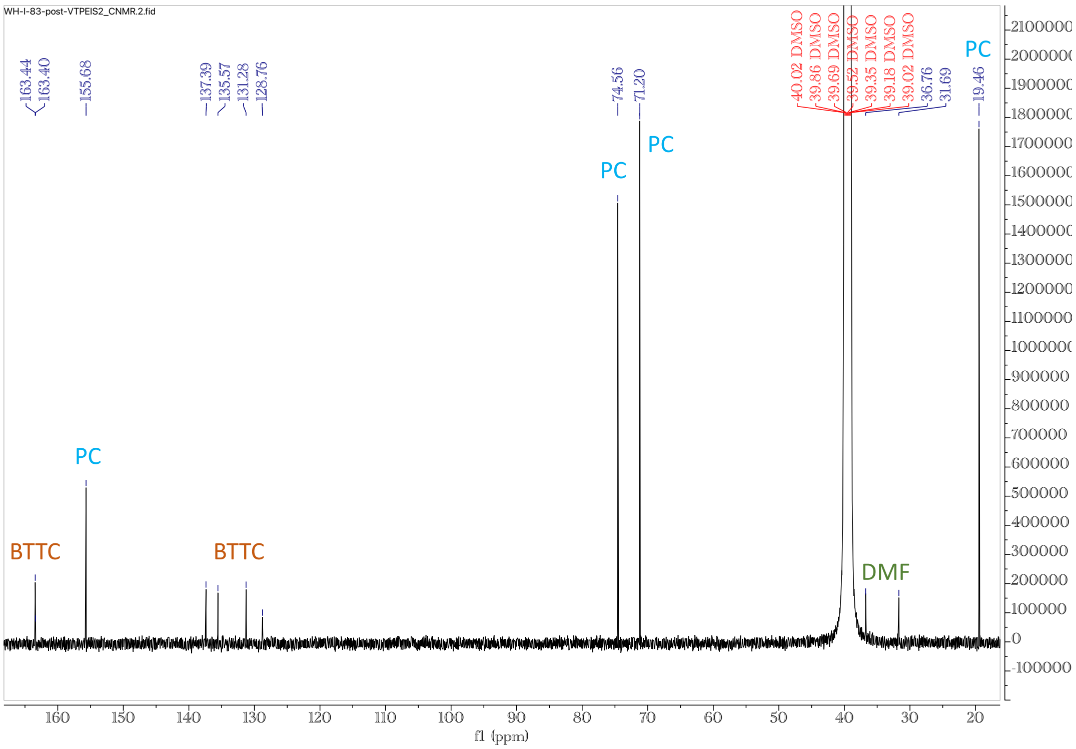

Figure S11. ^{13}C NMR of digested LiCuBTTC (175% Li^{+})-PC in dimethyl sulfoxide-d_6.

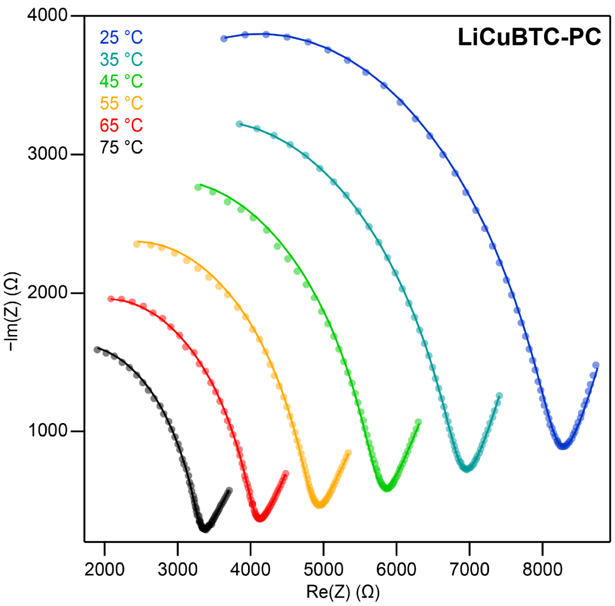

Figure S12. Variable-temperature potentiostatic electrochemical impedance spectroscopy (VT-PEIS) of LiCuBTC-PC.

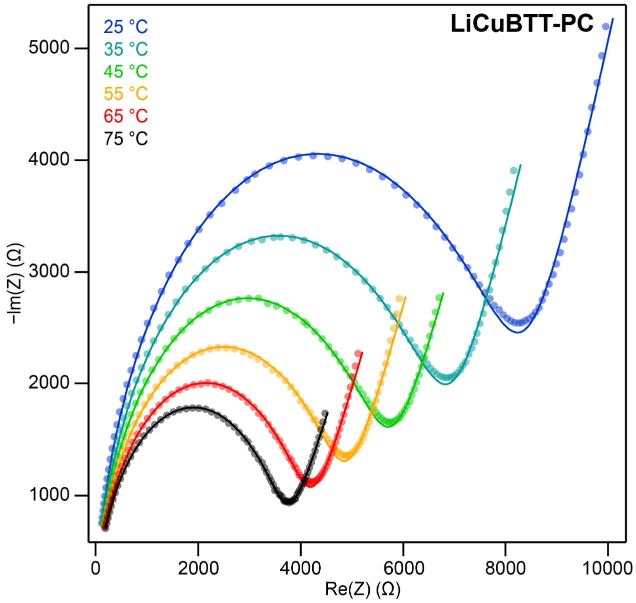

Figure S13. Variable-temperature potentiostatic electrochemical impedance spectroscopy (VT-PEIS) of LiCuBTT-PC.

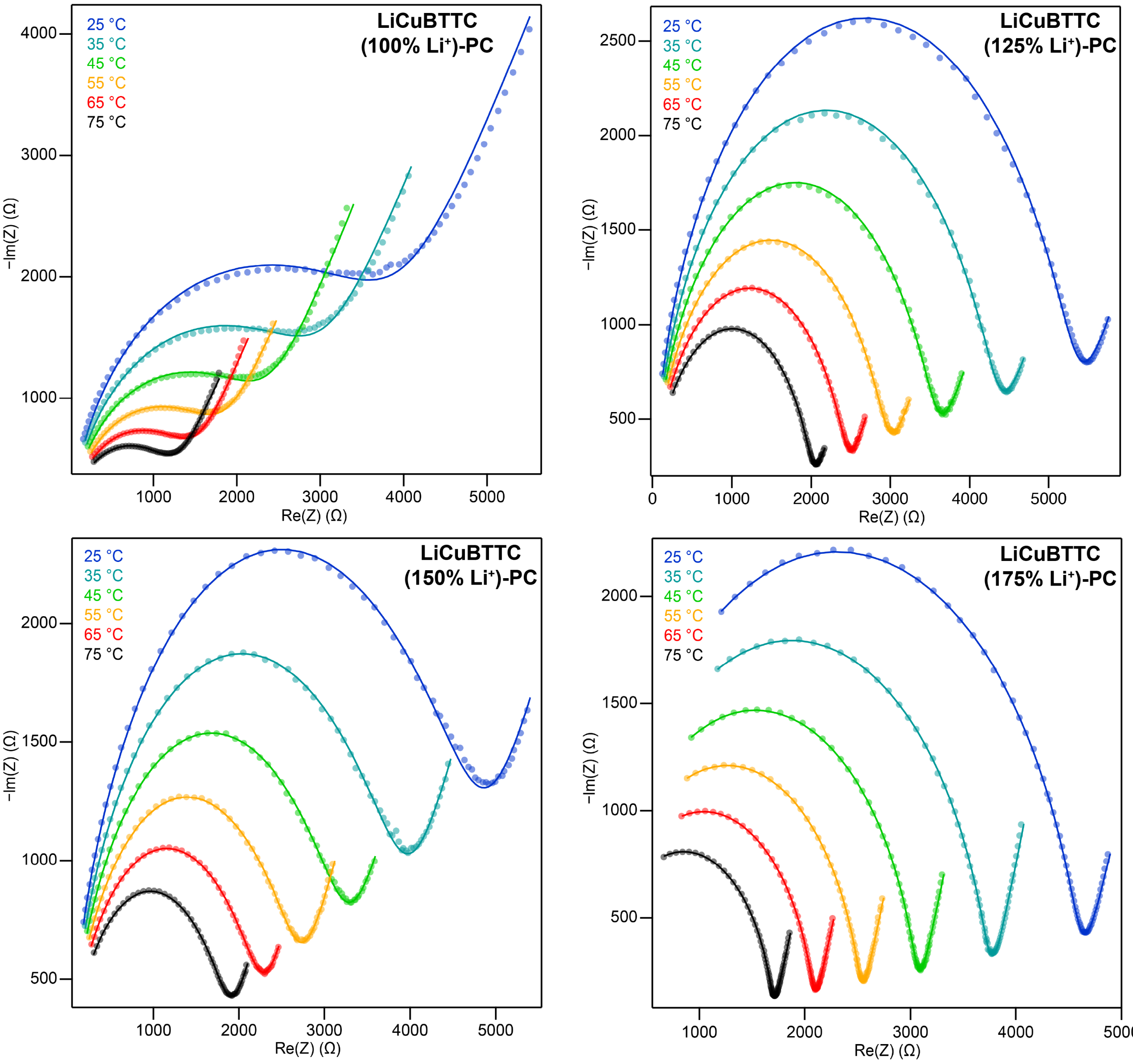

Figure S14. Variable-temperature potentiostatic electrochemical impedance spectroscopy (VT-PEIS) of LiCuBTTC-PC.

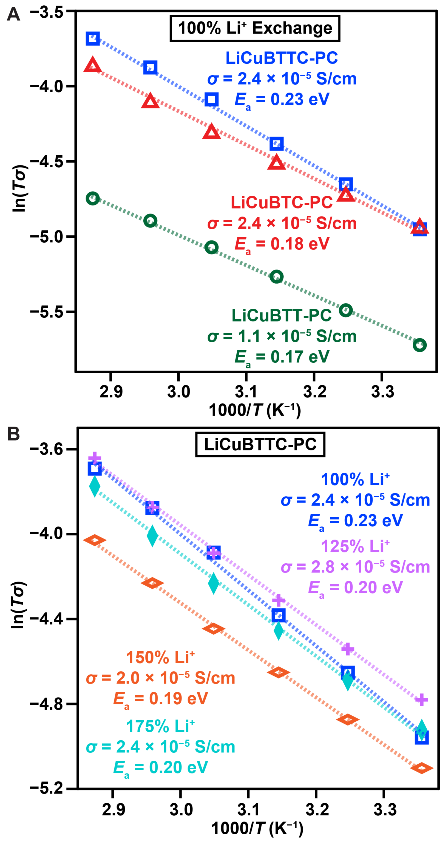

Figure S15. ln($T\sigma$) versus 1000/T Nernst-Einstein plots of (A) materials with quantitative Li^+ exchange and (B) LiCuBTTC with different Li^+ loadings, where T is temperature and σ is the conductivity. The linear fits are shown as the dashed lines. The Li^+ conductivities (σ) at 25 °C and the activation energies (E_a) are shown.

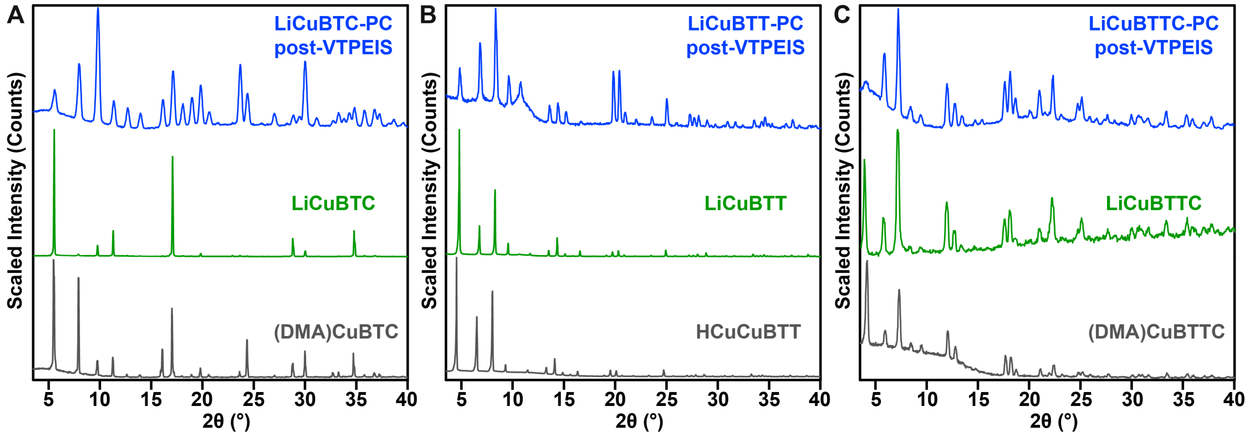

Figure S16. Powder X-ray diffraction (PXRD) patterns of (A) $[CuBTC]^{3-}$, (B) $[CuBTT]^{3-}$, and $[CuBTTC]^{3-}$ frameworks before Li^+-exchange (gray), after Li^+-exchange (green), and after electrochemical measurements (blue).

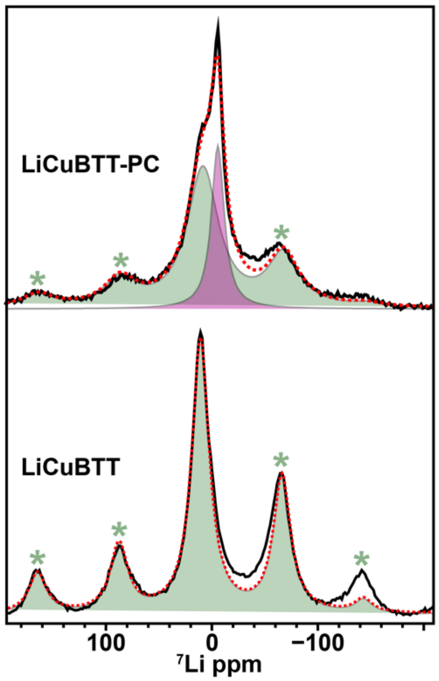

Figure S17. ^{7}Li solid-state NMR spectra of activated LiCuBTT with and without propylene carbonate collected at 12 kHz magic-angle spinning (MAS) rate. The solid black and dashed red lines indicate the experimental and fitted spectra, respectively. The shaded areas under peaks represent the fits of individual peaks, and their spinning sidebands are indicated with asterisks.

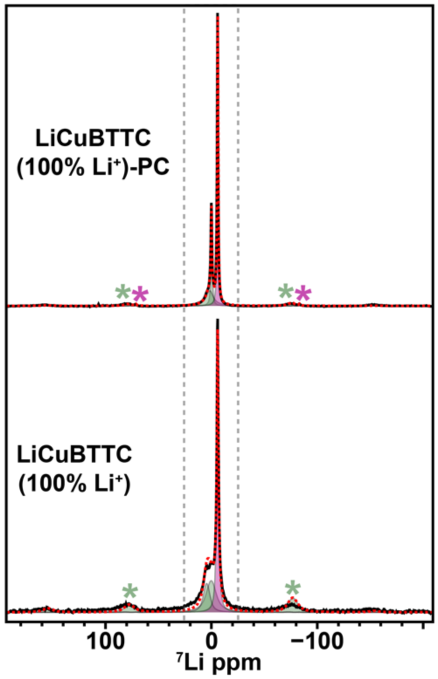

Figure S18. ^{7}Li solid-state NMR spectra of activated LiCuBTTC (100% Li^+) with and without propylene carbonate collected at 12 kHz magic-angle spinning (MAS) rate. The solid black and dashed red lines indicate the experimental and fitted spectra, respectively. The shaded areas under

peaks represent the fits of individual peaks, and their spinning sidebands are indicated with asterisks. The gray dashed lines act as the visual guide for the decrease in peak width after PC was added.

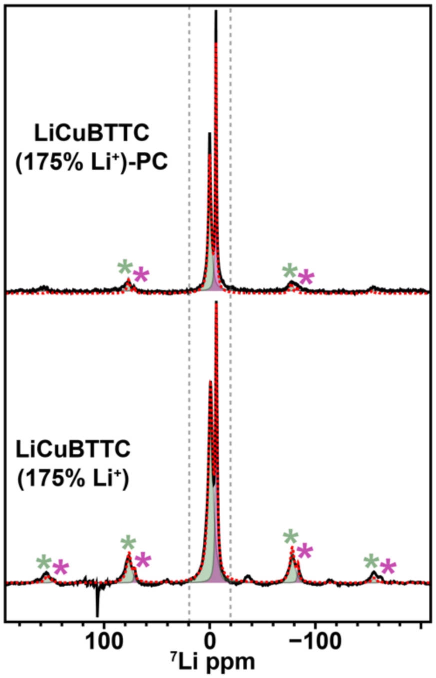

Figure S19. ^{7}Li solid-state NMR spectra of activated LiCuBTTC (175% Li^{+}) with and without propylene carbonate collected at 12 kHz magic-angle spinning (MAS) rate. The solid black and dashed red lines indicate the experimental and fitted spectra, respectively. The shaded areas under peaks represent the fits of individual peaks, and their spinning sidebands are indicated with asterisks. The gray dashed lines act as the visual guide for the decrease in peak width after PC was added.

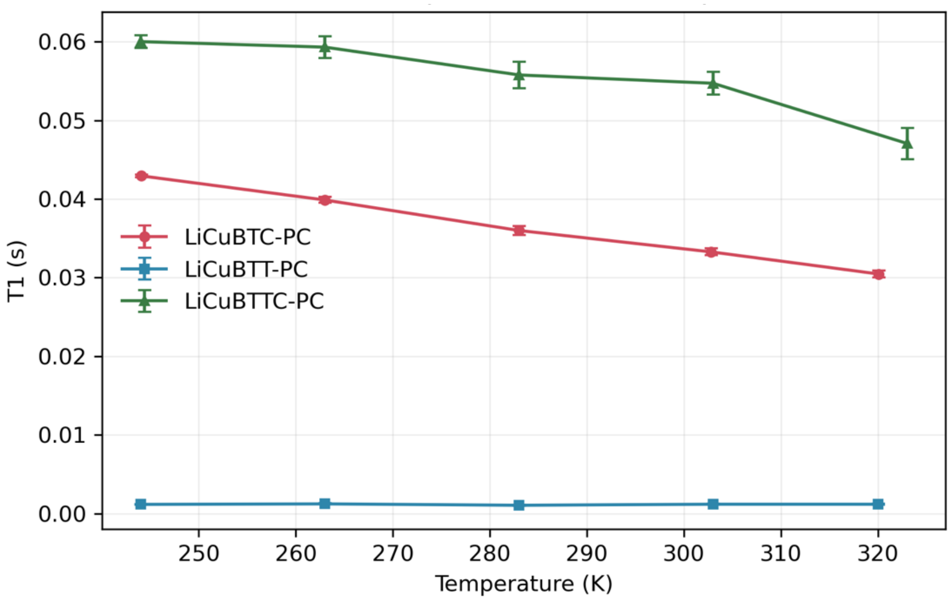

Figure S20. Temperature-dependence of the ^{7}Li spin-lattice relaxation time (T_1) of the major conducting Li^+ species in LiCuBTC-PC (red), LiCuBTT-PC (blue), and LiCuBTTC (100% Li^+)-PC (green).

Discussion regarding Figure S20:

LiCuBTC-PC and LiCuBTTC (100% Li^+)-PC exhibit a clear decrease in T_1 with increasing temperature, while LiCuBTT-PC shows nearly temperature-independent T_1 over the same range.

Within the paramagnetic relaxation enhancement (PRE) framework, the effective correlation time is given by $\tau_c^{-1} = \tau_r^{-1} + T_{1e}^{-1}$, where τ_r is the Li^+ motional correlation time and T_{1e} is the electron spin-lattice relaxation time of the Cu^{2+} centers. For Cu^{2+} coordination complexes, we estimate T_{1e} to be in the 1–5 ns range at room temperature from solid-state paramagnetic NMR and EPR studies (*J Am Chem Soc*. **2012**, *134*, 8693; *J. Phys. Chem. Lett.* **2017**, *8*, 5871.) For propylene carbonate-solvated Li^+ confined in MOF pores, variable-temperature ^{7}Li T_1 NMR measurements on a diamagnetic reference system yield Li^+ motional correlation time τ_r in the range of ~1-10 ns at room temperature (*ACS Appl. Mater. Interfaces* **2021**, *13*, 53986.).

This provides a semi-quantitative basis for interpreting our data. The two samples (LiCuBTC-PC and LiCuBTTC-PC) with temperature-dependent T_1 are consistent with $\tau_r \lesssim T_{1e}$ (~1–5 ns), placing their Li^+ dynamics in the regime where motional correlation contributes to relaxation — in line with their higher ionic conductivities (2.6×10^{-5} S/cm at 25 °C). LiCuBTT-PC with temperature-independent T_1 is consistent with $\tau_r \gg T_{1e}$, where relaxation is dominated by electron spin dynamics and Li^+ motion is slow — consistent with its lowest ionic conductivity among the three samples (1.1×10^{-5} S/cm at 25 °C). A fully quantitative extraction of absolute correlation times is not feasible without independent knowledge of T_{1e} for the specific $[(Cu_4Cl)_3L_8]^{3-}$ (where L^{3-} = linker) cluster geometry and the distribution of Li–Cu distances, but the semi-quantitative conclusions are robust across the plausible parameter range. The trends

across the isoreticular MOFs from the variable-temperature T_1 measurements are fully consistent with both the spinning sideband analysis and the measured conductivities, providing a convergent molecular-level picture of Li^+ transport.

References

1. Tan, Y. X.; He, Y. P.; Zhang, J. Pore partition effect on gas sorption properties of an anionic metal-organic framework with exposed Cu^{2+} coordination sites. *Chem. Commun.* **2011**, *47*, 10647.
2. Dincă, M.; Han, W. S.; Liu, Y.; Dailly, A.; Brown, C. M.; Long, J. R. Observation of Cu^{2+}-H_2 interactions in a fully desolvated sodalite-type metal-organic framework. *Angew. Chem. Int. Ed.* **2007**, *46*, 1419.
3. Liu, G.; Li, B. B.; Tan, Y. X.; Su, K.; Yuan, D. Comparative Stability & Sorption Study of Two the-Type Metal-Organic Frameworks with Different Multiplicate Metal-Ligand Interactions in Secondary Building Units. *Cryst. Growth Des.* **2017**, *17*, 418.
